# Oral Sodium Loading Test Is More Sensitive Than Seated Saline Infusion Test to Confirm Overt Primary Aldosteronism

**DOI:** 10.1210/jendso/bvae209

**Published:** 2024-11-26

**Authors:** Laurence Duquet, Laura Lefebvre, Samuel Lemaire-Paquette, Mandy Malick, Pierre-Luc Mallet, Matthieu St-Jean

**Affiliations:** Departement of Medecine, Centre Hospitalier de l’Université de Sherbrooke (CHUS), Sherbrooke, QC J1H 5N4, Canada; Departement of Medecine, Centre Hospitalier de l’Université de Sherbrooke (CHUS), Sherbrooke, QC J1H 5N4, Canada; Biostatistics Department, Research Center of the Centre Hospitalier de l’Université de Sherbrooke (CHUS), Sherbrooke, QC J1H 5N4, Canada; Departement of Medecine, Centre Hospitalier de l’Université de Sherbrooke (CHUS), Sherbrooke, QC J1H 5N4, Canada; Department of Laboratory Medicine, Biochemistry Division, Centre Hospitalier de l’Université de Sherbrooke (CHUS), Sherbrooke, QC J1H 5N4, Canada; Departement of Medecine, Centre Hospitalier de l’Université de Sherbrooke (CHUS), Sherbrooke, QC J1H 5N4, Canada; Division of Endocrinology, Centre Hospitalier de l’Université de Sherbrooke (CHUS), Sherbrooke, QC J1H 5N4, Canada

**Keywords:** overt primary aldosteronism, oral sodium loading test, seated saline infusion test

## Abstract

**Context:**

Primary aldosteronism (PA), a frequent but underdiagnosed cause of hypertension, is associated with a significant burden of cardiovascular and renal complications. Studies have reported divergent results regarding the diagnostic performance of seated saline infusion test (SSIT) and oral sodium loading test (OSLT), 2 confirmatory tests recommended by the Endocrine Society Clinical Practice Guidelines. To our knowledge, no study directly compared the results of SSIT and OSLT to diagnose overt PA.

**Objective:**

We assessed the diagnostic performance of SSIT and OSLT in a group of patients with hypertension and elevated screening aldosterone–renin ratio (ARR). The diagnostic standard was defined as hypertension with or without hypokalemia with an elevated screening ARR and at least 1 abnormal confirmation test including OSLT and SSIT.

**Methods:**

A monocentric retrospective study was conducted, including 87 patients with hypertension with a positive screening who underwent both SSIT and OSLT. A diagnostic performance analysis was conducted using urinary aldosterone at a threshold of 27 nmol/day as the criterion for OSLT, in comparison to a plasma aldosterone concentration (PAC) exceeding 140 pmol/L following the saline infusion.

**Results:**

A statistically significant difference in sensitivity was observed between OSLT and SSIT, with OSLT demonstrating superior performance (*P* = .025). The aforementioned test exhibited concordance in 59 cases (65.5%), indicating that these methods are not equivalent (McNemar test *P* = .036).

**Conclusion:**

OSLT demonstrated a significantly higher sensitivity for diagnosing overt PA in comparison with the SSIT in our cohort of patients with hypertension with an abnormal screening ARR.

Primary aldosteronism (PA) represents a frequent cause of secondary hypertension, with an increased prevalence observed as the severity of hypertension increases [1-3]. PA is defined as an aldosterone excess that is renin and partly potassium independent [4, 5]. Aldosterone excess results in sodium and water retention. which in turn leads to hypertension and kaliuresis. Consequently, 30% to 40% of patients with PA present with hypokalemia [1]. Given the continuous spectrum of severity with which abnormal aldosterone secretion presents, the phenotype of patients with PA is highly variable. This ranges from normotensive individuals to those with resistant hypertension [6]. Moreover, in comparison to essential hypertension, PA is associated with an increased risk of cardiovascular, cerebrovascular, and renal complications, regardless of blood pressure control [7-9].

According to the latest 2016 Endocrine Society guidelines for the management and diagnosis of PA, patients who meet the established screening criteria should undergo measurement of the aldosterone–renin ratio (ARR) [10, 11] and in the event of an elevated ARR, a confirmatory test should be conducted [12]. Four different confirmatory tests are currently recommended; the fludrocortisone suppression test, the oral sodium loading test (OSLT), the seated saline infusion test (SSIT), and the captopril challenge test (CCT) [11, 13, 14]. The objective of these tests is to reduce renin–angiotensin system (RAS) activity and consequently suppress aldosterone production by the zona glomerulosa cells of the adrenals. In the event of insufficient suppression of plasma aldosterone concentration (PAC) following RAS suppression, a diagnosis of overt PA can be made [11]. The aforementioned confirmatory tests represent a crucial diagnostic step in the identification of overt PA, as up to 30% to 50% of patients with an elevated ARR and a low renin will demonstrate appropriate suppression of PAC during these tests [15, 16]. Patients with hypertension with an elevated screening ARR and a low renin, but normal aldosterone suppression during confirmatory test, are considered to have a subtle form of PA and are typically managed with mineralocorticoid receptor antagonist [6]. Patients with confirmed overt PA should undergo adrenal veins sampling (AVS) to assess the lateralization of the disease and, if indicated, undergo unilateral adrenalectomy in case of significantly lateralized PA [6].

The current literature reveals divergent results regarding the effectiveness and relative reliability of the 4 confirmatory tests. The latest guidelines lack sufficient evidence to prioritize 1 test over another [11, 15, 17]. The fludrocortisone suppression test is often used as the gold standard for benchmarking other confirmatory tests [2, 10, 18]. However, this procedure is frequently overlooked due to the risk of inducing hypokalemia, worsening hypertension, and its significant costs to the healthcare system [14, 16, 19]. Currently, the SSIT and CCT are the most commonly utilized confirmatory tests [20]. The PAC thresholds and assays employed to quantify PAC vary between centers. Additionally, many centers rely on the result of a single confirmatory test to confirm or exclude overt PA [20].

The OSLT requires administration of an oral sodium load to achieve a 24-hour urinary sodium excretion of at least 170 mmol per day. The objective of the test is to expand intravascular volume and subsequently physiologically suppress the RAS activity. Due to the unreliability of single measurements of PAC, caused by its variable and pulsatile secretion in PA and high-sodium states, 24-hour urinary aldosterone excretion is used as a consistent and integrated measure of renin-independent aldosterone production [1, 21]. Many centers rely primarily on OSLT as their confirmatory test, given that it can be conducted on an outpatient basis [22]. Data on the comparative diagnostic performance of OSLT and SSIT are limited [11, 23]. In patients with resistant hypertension, studies using the saline infusion test as the confirmatory test have reported an overt PA a prevalence of approximately 7% [11, 24, 25], compared to 22% in a recent American cohort in which overt PA diagnosis was made using OSLT [1]. It would be of interest to ascertain whether there is a difference in diagnostic performance between OSLT and SSIT, as this could help to elucidate the significant discrepancy in the prevalence of overt PA observed between studies.

## Materials and Methods

### Study Design

A monocentric retrospective cohort study was conducted at the Centre Hospitalier de l’Université de Sherbrooke (CHUS) in Sherbrooke, Canada. The study included patients who were followed at our center between July 2018 and February 2024. A comprehensive review of individual medical records was conducted for all patients included in the study. A database was established through the implementation of a systematic probabilistic sampling methodology. The data pertaining to the patients were extracted manually from their institutional electronic medical records (ARIANE), which encompassed both outpatient and inpatient notes, as well as follow-up visits.

The project received approval from the research ethics committee of the Centre intégré de santé et des services sociaux de l'Estrie-Centre Hospitalier Universitaire de Sherbrooke (CISSS-CHUS), with a delegated consent process (Project#2023-4928).

### Study Objectives

The primary objective of this study is to assess the diagnostic efficacy of OSLT and SSIT in a cohort of patients with an abnormal ARR screening test (including a low direct renin concentration [DRC]), employing diagnostic criteria of overt PA in accordance with the current standard of care. The diagnostic reference standard was defined as the presence of clinical hypertension with or without hypokalemia, an abnormal screening ARR (including a DRC less than 8 ng/L) and at least 1 abnormal confirmatory test (OSLT or SSIT) using thresholds described in “Confirmatory Tests Modalities.”

Secondary objectives included the analysis of the concordance rate of the SSIT and OSLT results and the evaluation of clinical, biochemical, and radiological characteristics to determine whether they were predictive of having an abnormal OSLT or SSIT result. The characteristics assessed for predictive value in regard to abnormal responses to the aforementioned tests included age, sex, body mass index, lowest serum potassium level, oral potassium supplement intake, blood pressure prior to the SSIT, number and class of antihypertensive drugs (including the calculated defined daily dose [DDD]), PAC, DRC, screening ARR, computed tomography adrenal imaging findings and AVS results.

### Population

The study population consisted of all patients aged 18 years and older with a screening ARR of ≥60 pmol/ng (PAC/DRC) and who underwent both OSLT and SSIT and were followed at CIUSSS de l'Estrie-CHUS. At our institution, a single endocrinologist was primarily responsible for evaluating patients for potential PA. The inclusion criteria were as follows: the patient must have been at least 18 years of age, exhibited an abnormal screening ARR (≥60 pmol/ng), and undergone both SSIT and OSLT within a period of less than 12 months. The exclusion criteria included relative contraindications to sodium overload, such as uncontrolled severe hypertension, advanced chronic kidney disease (stage IV or V), heart failure, significant cardiac arrhythmias, and severe uncontrolled hypokalemia. Pregnant women were also excluded from the study.

### ARR Screening Modalities

In our center, patients are typically instructed to withhold spironolactone, eplerenone, oral contraceptive pills, and estrogenic hormonal replacement therapy for a minimum of 4 weeks prior to undergoing screening ARR and confirmatory tests. The other antihypertensive drugs are typically maintained and continued, as prior studies have demonstrated that they have no significant impact on the results of the diagnosis of PA when the DRC is suppressed (<8 ng/L) [6]. In patients with a high pretest probability of overt PA (such as selected patients in our cohort), a single normal ARR with unsuppressed renin does not necessarily exclude PA. In the event that the DRC is >8 ng/L and/or the ARR was below the threshold of 60 pmol/ng, the ARR measurement is usually repeated after discontinuation of any potentially confounding antihypertensive medication for at least 14 days. In our institution, we utilize an ARR cutoff of 60 pmol/ng to enhance sensitivity [11]. Following an abnormal screening ARR, we frequently request the patients to perform both the OSLT and the SSIT in parallel during a short period of time to confirm an overt PA diagnosis. In other cases, patients have been evaluated by another specialist and referred to the clinic with a normal or near normal confirmatory test already performed, and in the case of a high pretest probability, another confirmatory test was ordered (ie, OSLT if previously performed SSIT was normal).

### Confirmatory Tests Modalities

Before we do the confirmatory tests, we make sure that serum potassium is under control. For the OSLT, patients were instructed to ingest 6 g of sodium chloride (NaCl) orally for a period of 3 consecutive days and to subsequently perform their 24-hour urine collection. The result was deemed to be abnormal when the urinary aldosterone exceeded 27 nmol/day [26]. Sodium intake adequacy was confirmed by daily urinary sodium excretion and patients were excluded (n = 11) if their daily urinary sodium was less than 170 mmol/day and if they declined to repeat the test with a larger sodium load (8 g of NaCl/day for 3 days). To further assess the 24-hour urine collection, we considered the creatinine urinary excretion, which was required to be included in the reference intervals. The normal range for females was established to be between 5 and 15 mmol/day, while the normal range for males was established to be between 8 and 25 mmol/day.

For the SSIT, patients were required to remain seated for a minimum of 30 minutes prior to the baseline venipuncture, which was used to measure the baseline PAC and DRC. Subsequently, 2 L of 0.9% NaCl was administered intravenously over a period of 4 hours (500 mL/hour), after which PAC and DRC were reassessed. The SSIT was deemed abnormal if the postinfusion PAC exceeded 140 pmol/L [11]. Throughout the duration of the test, patients were instructed to remain seated.

### Clinical and Biochemical Parameters

The following data were obtained from the patient's electronic medical records: age, sex, body mass index, lowest serum potassium level, oral potassium supplement intake, blood pressure before the SSIT, number and class of antihypertensive drugs (including the calculated DDD), PAC, DRC, screening ARR, computed tomography adrenal imaging findings, and SSIT (including PAC and DRC at baseline and at the end of the infusion) and OSLT results (including 24 hours urinary aldosterone, sodium and creatinine levels). The calculated DDD provides a standard measurement allowing for an independent comparison of the prescribed antihypertensive drugs between the patients [27]. Furthermore, if available, we evaluated the results of AVS.

In our center, AVS is conducted by a single interventional radiologist. AVS was conducted with bilateral simultaneous sampling of both adrenal veins and the inferior vena cava before and after a 250-µg intravenous bolus of cosyntropin as previously described in a paper published by the Lacroix team [28]. AVS was performed in selected patients (n = 27; 48% of PA cases) and PA was considered lateralized if basal lateralized ratio was >4 and/or the post-adrenocorticotropin (ACTH) lateralized ratio was >4 [29, 30].

### Biochemical Assays

Serum and EDTA plasma samples were collected respectively for PAC and DRC analysis to assess the ARR. A 24-hour urine collection was performed for urinary aldosterone and sodium daily output quantification. The PAC and urine aldosterone were quantified by liquid chromatography coupled to tandem mass spectrometry (LC-MS/MS) (ie, Agilent 1290 and 6460). The LC-MS/MS method was implemented in the clinical laboratory as a laboratory-developed test (ie, LDT) and validated based on Canadian Standard Association and the Clinical and Laboratory Standards Institute guidelines to fulfill ISO15189 criteria requirement. The plasma DRC was analyzed by 2 distinct methodologies; initially, by enzyme-linked immunosorbent assay (ELISA) (ie, RENIN-ELISA/Active Renin, Cisbio assays) until January 2020, subsequently by chemiluminescent immunoassay (ie, LIAISON XL Direct Renin, DiaSorin). The electrolytes (ie, serum potassium and urinary sodium) were analyzed by indirect ion-selective electrodes on a Roche modular/Cobas platform. All tests were conducted at the Laboratory Medicine Department of the ISO15189 accredited CIUSSS-CHUS Fleurimont's Hospital (Sherbrooke, CA).

### Statistical Analysis

Dichotomous and nominal variables are described using frequencies and percentages, whereas continuous variables are presented as mean (SD) or medians (interquartile range) based on their distribution. Any missing data are reported where applicable. The analysis is conducted using a significance threshold of 5%. SPSS v.28 software was used to compute the results.

The primary objective, which is the diagnostic performance of both OSLT and SSIT for overt PA, is assessed using sensitivity, specificity, negative predictive value (NPV), and positive predictive value (PPV) at the previously mentioned thresholds. The diagnostic parameters are reported with a 95% CI and compared using corresponding Z scores and *P* values.

For the secondary objectives, a cross-tabulation table was presented, displaying the proportions of concordance and discordance along with 95% CIs. A McNemar test was employed to ascertain whether a significant discrepancy existed between the positivity rates observed for the OSLT and SSIT. Moreover, patients were classified into 3 groups based on the results of sodium overload tests. The first group comprised patients who exhibited abnormal results exclusively during SSIT. The second group consisted of patients who exhibited isolated abnormal results on the OSLT. The third group consisted of patients who exhibited abnormal results on both tests (both abnormal [BA]). To evaluate the characteristics of independent subgroups and assess for potential differences in baseline demographic characteristics and potential predictive factors, a paired analysis of the subgroup characteristics was conducted.

Associations were also evaluated using unpaired Student's t-tests or Mann–Whitney's U tests to compare means or rank, and χ^2^ tests (Fisher exact) to compare proportions. Corresponding *P* value are also presented. No *P* value adjustments were made since those analyses are considered as exploratory.

## Results

### Patient Selection and Demographic

**Figure 1.**
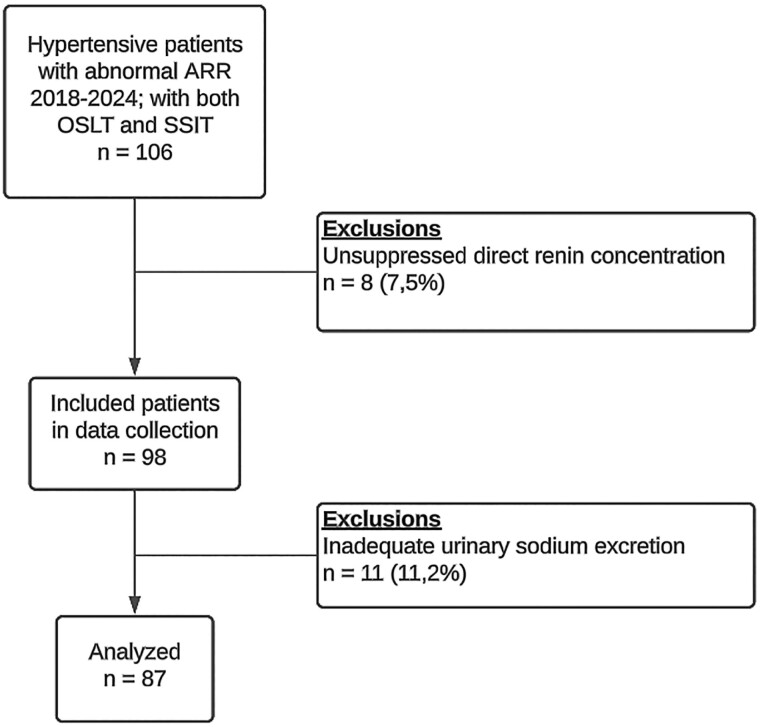
Flow diagram of patients included in the retrospective study.

Initially, 106 patients met the inclusion criteria (Fig. 1). Eight patients were excluded based on the presence of unsuppressed basal DRC during SSIT (DRC > 8 ng/L, ranging from 15.2 to 81.3 ng/L), which is suggestive of a renin-dependent aldosterone excess. Eleven patients with urinary aldosterone greater than 27 nmol/day were excluded from the final analysis because of inadequate urinary sodium excretion (<170 mmol/day) and unwillingness to repeat the test. A second OSLT was performed in 12 patients (14% of the total cohort) due to insufficient sodium intake. The analysis included negative OSLT (urinary aldosterone <27 nmol/day), even when the urinary sodium excretion was <170 mmol/day (n = 9; with urinary sodium excretion ranging from 66 to 164 mmol/day). Patient characteristics of the overall cohort and confirmed overt PA cases are detailed in Table 1.

**Table 1.** Characteristics of patients of the overall cohort (n = 87) and patients with confirmed primary aldosteronism (PA) (n = 54)

	Overall cohort	Confirmed PA
Age (years) mean ± SD	54 ± 14	56 ± 13
Sex, n (%)		
Male	28/87 (32)	23/54 (43)
Female	59/87 (68)	31/54 (57)
ARR baseline (pmol/ng), mean ± SD	181.0 ± 127.0	215.9 ± 145.0
Aldosterone (pmol/L)	392.8 ± 222.5	452.1 ± 220.6
Direct renin concentration (ng/L)	3.0 ± 2.4	3.0 ± 2.4
BMI (kg/m^2^), mean ± SD	31.5 ± 7.4	31.8 ± 6.6
Kalemia (mmol/L), mean ± SD	3.9 ± 0.3	3.8 ± 0.3
History of hypokalemia, n (%)	28/87 (32)	22/54 (41)
BP (mm Hg), mean ± SD		
Systolic	139 ± 18	143 ± 19
Diastolic	84 ± 10	85 ± 11
Number of antihypertensive drugs, mean ± SD	1.8 ± 1.5	2.3 ± 1.5
DDD baseline, mean ± SD	2.4 ± 2.2	3.2 ± 2.3

Confirmed PA defined by at least 1 abnormal test (SSIT and/or OSLT).

Data are expressed as mean ± SD or proportion of patients (percentage).

Abbreviations: BMI, body mass index; BP, blood pressure; DDD, defined daily dose.

### Diagnostic Tests Sequence

Both confirmatory tests were ordered simultaneously in 58% (51/87) of the patients included in the final analysis. In 28% (24/87) of the patients, the OSLT was ordered in patients with a high diagnostic probability and a near normal or normal SSIT that had been performed by another specialist before the patient was referred to our clinic. In 14% (12/87) of the final cohort, the SSIT was requested in patients who had a near normal or normal OSLT performed prior to referral to our clinic. Most patients completed both tests within a relatively short time frame. 47% (41/87) of patients completed both tests within the same month, 62% (54/87) within less than 2 months, 75% (65/87) within less than 3 months, and 92% within less than 6 months (80/87). The primary causes of these postponements were the necessity to repeat the OSLT (due to inadequate sodium loading) or the delay in obtaining an appointment at our testing facility to conduct the SSIT.

### Antihypertensive Drug Regimen

The antihypertensive treatment regimen was identical for each patient during both confirmatory tests. The antihypertensive treatment regimen taken by the patients in the different groups based on their confirmatory test result is shown in Table 2. We have also described in Table 2 the antihypertensive regimen taken by the patient who did not achieve sufficient daily urinary sodium excretion.

**Table 2.** Antihypertensive drugs regimen of each group

	SSIT abnormal	OSLT abnormal	Both test abnormal	Both test normal	Excluded due to insufficient 24-hour sodium urinary excretion
	n = 8	n = 20	n = 26	n = 33	n = 11
Potassium-sparing diuretics	2 (25%)	8 (40%)	12 (46%)	2 (6%)	3 (37%)
ACE inhibitor or ARB	5 (62%)	8 (40%)	15 (58%)	9 (27%)	6 (75%)
DHP CCB: amlodipine	6 (75%)	9 (45%)	16 (61%)	9 (27%)	6 (75%)
Non-DHP CCB	0 (0%)	3 (15%)	4 (15%)	3 (9%)	5 (62%)
Beta-adrenergic blockers	1 (12%)	6 (30%)	6 (23%)	8 (24%)	5 (62%)
Hydralazine	0 (0%)	2 (10%)	2 (8%)	0 (0%)	0 (0%)
Alpha-1 blocker	0 (0%)	3 (15%)	5 (19%)	0 (0%)	2 (25%)
Thiazide	2 (25%)	4 (20%)	3 (11%)	0 (0%)	2 (25%)
Loop diuretic	0 (0%)	0 (0%)	0 (0%)	1 (3%)	0 (0%)
Central alpha-2 agonist	1 (12%)	0 (0%)	0 (0%)	0 (0%)	0 (0%)

Data are expressed as proportion of patients (%).

Abbreviations: ACE, angiotensin-converting enzyme; ARB, angiotensin type II receptor blocker; CCB, calcium channel blocker; DHP, dihydropyridine; OSLT, oral salt loading test; SSIT, seated saline intravenous test.

### Diagnostic Performance of SSIT Compared to OSLT

Based on the reference diagnostic criteria, OSLT yielded a sensitivity of 84.9%, specificity of 100%, a PPV of 100%, and a NPV of 61.9%. SSIT had a sensitivity of 64.2%, specificity of 100%, a PPV of 100%, and a NPV of 40.6%. As shown in Table 3, a statistically significant difference in sensitivity was noted between the 2 tests in favor of the OSLT (*P* = .025).

**Table 3.** Comparison of diagnostic performance of seated saline infusion test (SSIT) and oral sodium loading test (OSLT)

	Abnormal SSIT	Abnormal OSLT	*P* value
	95% CI	95% CI	
Sensitivity (%)	64.2 (51.2-77.1)	84.9 (75.3-94.5)	.025
Specificity (%)	100.0 (77.2-100.0)	100.0 (77.2-100.0)	1.000
NPV (%)	40.6 (25.2-57.7)	61.9 (40.9-79.3)	.217
PPV (%)	100.0 (89.8-100.0)	100.0 (92.1-100.0)	1.000

*P* values calculated by using a Z score.

Abbreviations: NPV, negative predictive value; PPV, positive predictive value.

### Concordance Rate Analysis

As shown in Table 4, the positive concordance between SSIT and OSLT was only 67.8% (95% CI 58.0-77.6), indicating a significant discrepancy between the 2 tests (McNemar's test *P* = .036). In 8 patients (9.2%) the SSIT was abnormal, but the OSLT was not. Similarly, 20 patients (23.0%) had an abnormal OSLT with a normal SSIT.

**Table 4.** Mann–Whitney U test baseline demographic, biochemical and radiological characteristics comparison statistical significance between abnormal seated infusion test (SSIT) and abnormal oral salt loading (OSLT)

	SSIT abnormal	OSLT abnormal	*P* value
	n = 8	n = 20	
Age (years), mean ± SD	61.7 ± 14.6	51.0 ± 12.3	.056
Sex, n (%)			
Male	2 (25%)	6 (30%)	1.000
Female	6 (75%)	14 (70%)	
BMI (kg/m^2^)	31.3 ± 4.4	30.9 ± 7.0	.308
Number of antihypertensive drugs, mean ± SD	2.1 ± 1.1	2.2 ± 2.0	.734
DDD baseline, mean ± SD	2.6 ± 1.3	3.1 ± 3.1	.980
Kalemia (mmol/L)	3.9 ± 0.2	3.8 ± 0.2	.322
ARR baseline (pmol/ng), mean ± SD	156.6 ± 121.8	184.4 ± 128.9	.542
Aldosterone (pmol/L)	403.6 ± 227.7	499.1 ± 276.2	.162
Direct renin concentration (ng/dL)	3.5 ± 2.8	3.7 ± 2.8	.858
Baseline SSIT results, mean ± SD			
Aldosterone before SSIT (pmol/L)	373.9 (182.0)	322.7 (227.0)	.401
ARR before SSIT (pmol/ng)	147.4 (166.0)	136.9 (154.1)	.899
Unilateral on AVS, n (%)	1/3 (33%)	6/8 (86%)	.491
Adrenal adenoma, n (%)	3/6 (50%)	3/17 (18%)	.279

Data are expressed as mean ± SD or proportion of patients (%).

Unpaired t tests or Mann–Whitney U tests to compare means or medians, and χ^2^ tests or Fisher exact tests to compare proportions.

Abbreviations: AVS, adrenal vein sampling; BMI, body mass index; BP, blood pressure; DDD, defined daily dose.

### Biochemical and Radiological Predictors of Abnormal Result During OSLT, SSIT, or BA Tests

No statistically significant difference was observed between the baseline demographic characteristics of the OSLT, SSIT and BA groups. However, there was a trend toward an older age in the SSIT abnormal group (61.8 ± 14.6 years) compared with the OSLT (51.0 ± 12.3 years; *P* = .056). A similar trend is observed when comparing the OSLT and BA groups (58.2 ± 12.6 years; *P* = .052). Compared with the group with BA tests, there was also a trend toward a higher proportion of women in the group with an isolated abnormal OSLT (*P* = .062) (Tables 4 and 5).

**Table 5.** Mann–Whitney U test baseline demographic characteristics comparison statistical significance between abnormal seated saline infusion test (SSIT), abnormal oral salt loading (OSLT) and both abnormal (BA) test groups

	Both SSIT and OSLT abnormal	*P* value abnormal SSIT vs BA	*P* value abnormal OSLT vs BA
	n = 26		
Age (years), mean ± SD	58.2 ± 12.6	.416	.052
Sex, n (%)			
Male	11 (42%)	.225	.062
Female	15 (58%)		
BMI (kg/m^2^), mean ± SD	32.9 ± 6.9	.910	.228
Number antihypertensive drugs, mean ± SD	2.4 ± 1.2	.527	.263
DDD baseline, mean ± SD	3.3 ± 1.8	.319	.393
Kalemia (mmol/L), mean ± SD	3.8 ± 0.4	.206	.396
ARR baseline (pmol/ng), mean ± SD	258.5 ± 155.0	.113	.111
Aldosterone (pmol/L)	430.9 ± 166.3	.417	.756
Direct renin concentration (ng/dL)	2.3 ± 1.7	.110	.047
Baseline SSIT results, mean ± SD			
Aldosterone before SSIT (pmol/L)	559.4 ± 457.0	.291	.015
ARR before SSIT (pmol/ng)	221.4 ± 187.4	.465	.090
Unilateral on AVS, n (%)	9/16 (56%)	.582	.657
Adrenal adenoma, n (%)	12/24 (50%)	1.000	.034

Data are expressed as mean ± SD or proportion of patients (percentage).

Unpaired t tests or Mann–Whitney U tests to compare means or medians, and χ^2^ tests or Fisher exact tests to compare proportions.

Abbreviations: AVS, adrenal vein sampling; BMI, body mass index; BP, blood pressure; DDD, defined daily dose.

The baseline DRC (mean ± SD) in the screening ARR was significatively lower in the BA group than in the isolated OSLT abnormal group (2.3 ± 1.7 vs 3.8 ± 2.8 ng/dL; *P* = .047). Baseline PAC (mean ± SD) during the SSIT was higher in the BA group than in the OSLT group (559.4 ± 457.0 vs 322.7 ± 227.0 pmol/L; *P* = .015). When comparing the BA and OSLT groups, there is a trend toward a higher initial baseline ARR (mean ± SD) during the SSIT in the BA group (221.4 ± 187.4 vs 136.9 ± 154.1 pmol/ng; *P* = .090).

The incidence of adenomas observed on CT imaging was significantly lower in the OSLT group (17.6%, 3/17) than in the BA group (50%, 12/24) (*P* = .034).

### PA Lateralization

AVS was performed in a subset of patients with confirmed overt PA (n = 27/54, 50%) to assess lateralization. Of these 27 patients, 16 (59%) demonstrated significant lateralization of aldosterone production based on AVS results. There was no significant difference in the rate of significant lateralization between the SSIT, the OSLT, or the BA groups (Tables 4 and 5).

## Discussion

The existing literature on the comparative performance of OSLT and SSIT for the diagnosis of overt PA is limited. However, since both techniques are widely used, a comparison of their diagnostic performance is relevant [15, 17, 23]. In our cohort of patients with hypertension with an abnormal ARR screening test, low DRC, and at least 1 positive test using thresholds in accordance with the current standard of care for the diagnosis of overt PA [11, 22, 31], the OSLT demonstrated a higher diagnostic sensitivity than the SSIT. In addition, 32.2% (n = 28) of the cohort had a notable discrepancy between OSLT and SSIT results, with only 8 patients in whom SSIT was the only abnormal confirmatory test. Overall, these results suggest that the OSLT detected more overt PA cases than the SSIT in our cohort.

The higher diagnostic performance of OSLT may be a consequence of its integration of aldosterone fluctuations over a 24-hour period. This longer time integration may be more sensitive for the diagnosis of PA than the evaluation of PAC changes over only a 4-hour period, as done during the SSIT. In patients with PA, PAC has previously been shown to be regulated by several aberrant stimuli that regulate plasma aldosterone, namely mixed meals (glucose-dependent insulinotropic polypeptide), postural changes (vasopressin or catecholamines), ACTH, 5-HT4 receptor agonists, and gonadotropins [32]. As reported, an average of 4 abnormal responses are found in patients with PA [32]. One of the potentially significant contributors to the highest sensitivity associated with the OSLT is postural change, occurring frequently during the day. Compared with the supine position, the seated position has previously been shown to significantly improve the sensitivity of the saline infusion test, highlighting the substantial impact of postural changes on the sensitivity of the test [19]. Integrating all of these dysregulations of aldosterone secretion over a 24-hour period may explain the difference in diagnostic sensitivity we observed between the OSLT and the SSIT.

Another possible explanation for our findings is that aldosterone production appears to be pulsatile throughout the day and has a circadian fluctuation in healthy patients [33]. This concept might be observed in patients with PA, although perhaps aberrantly. In 1 patient with lateralized PA, the nadir for aldosterone levels was around midnight and the peak was around awakening [33, 34]. This finding supports that ACTH stimulates aldosterone production in some patients with PA [33, 34]. Some research also supports that in PA, aldosterone levels not only vary throughout the day, but also exhibit burst-like fluctuations, resulting in a wide range of PAC fluctuations of up to a 4-fold elevation [35]. The fact that aldosterone production appears to be pulsatile and to decrease significantly in the afternoon may explain the reduced sensitivity of SSIT, which relies on a single PAC measurement at the end of the infusion, typically in the late morning or early afternoon. In addition, many studies highlight the unreliability of relying solely on single PAC measurements, given the 22% to 25% intraindividual variability observed between 2 measurements, which may also contribute to the lowest diagnostic sensitivity observed with SSIT [11, 13, 26, 36].

Among the 28 patients with only 1 abnormal confirmatory test, no significant differences in biochemical and demographic factors were found, making it difficult to tailor a test to patients based on their baseline clinical and biochemical characteristics. However, there was a tendency for OSLT to be abnormal in younger patients compared to SSIT and patients with both tests abnormal. Also, the group with abnormal OSLT has slightly higher DRC on the screening ARR and lower PAC and ARR on the baseline measurement during SSIT than patients who have both SSIT and OSLT abnormal. These findings suggest that OSLT may identify overt PA earlier in the course of the disease and potentially milder forms of overt PA. There was also a trend for a greater proportion of women than men to have only abnormal OSLT compared with isolated abnormal SSIT and BA groups. A previous study by Pizzolo et al reported that a significantly higher proportion of hypertensive women with an elevated ARR have normal PAC suppression after saline infusion testing compared with men [37]. They conclude that women have more falsely elevated screening ARR than men, but the direct renin concentration of these women was low, suggesting some degree of renin-independent aldosterone excess. Therefore, OSLT may be a better confirmatory test in women than SSIT, but this needs to be confirmed in future studies. Also, fewer unilateral adenomas were detected on computed tomography imaging in the OSLT abnormal group (17.6%, 3/17) compared with the BA group (50% or 12/24, *P* = .034). In addition, a significant proportion of patients in the OSLT abnormal group (6/8) had significant lateralization on AVS, and 5 of them had no adrenal adenoma detected on computed tomography. This observation may be explained by an earlier detection of overt PA cases with OSLT, and potentially smaller adenomas may have been missed by the radiologist on computed tomography. It also confirms, as previously reported [4], that there is a poor correlation between observed unilateral adenomas and AVS lateralization in overt PA.

Although the OSLT was found to be more sensitive, it is not perfectly sensitive for diagnosing overt PA, as 8 out of 54 confirmed cases of PA (14%) were missed by the OSLT. We do not have a clear explanation for this finding. However, if the index of suspicion is high, the use of a second confirmatory test (ie, SSIT, CCT, or the upright posture stimulation test) seems appropriate.

We believe that patients with hypertension with a high screening ARR and a low DRC who have an abnormal OSLT and/or SSIT truly have PA. However, the confirmatory testing thresholds used in our cohort, which to our knowledge are the lowest used in clinical practice, are not sufficient to exclude PA. In fact, PA is currently conceptualized as a continuum of renin-independent aldosterone excess [6]. Nevertheless, confirmatory tests are recommended to determine which patients should undergo AVS and, in the case of a lateralizing disease, which patients should undergo unilateral adrenalectomy. Therefore, if the SSIT had been used exclusively in our cohort, a significant number of individuals with lateralizing disease who could have benefited from unilateral adrenalectomy would have been deprived of this potential treatment option.

It is important to note that OSLT and SSIT are contraindicated in patients at risk of developing complications related to sodium and volume overload. These patients were excluded from the present study. Nevertheless, most patients studied for PA are not at high risk of complications during SSIT or OSLT. However, in patients at risk of complications, CCT and the recently described upright position [38] are safer options for confirming overt PA diagnosis. Although some centers favor CCT over intravenous or oral sodium overload for PA confirmation, the diagnostic performance of these tests is not consistent across studies [11, 15, 17]. In addition, a recent study by Younes et al suggested that the upright posture test could serve as an additional diagnostic tool in patients with a high pretest probability of PA and a normal SSIT result [38]. A comparison of the diagnostic performance of the upright posture test with the OSLT in patients with a high pretest probability of PA would be of interest.

We do not think that the use of angiotensin type II receptor blockers, angiotensin-converting enzyme inhibitors, and diuretics, which have the potential to increase sodium excretion, explained the discrepancy of diagnostic performance that we observed between OSLT and SSIT. The proportion of patients taking these drugs was similar in all 3 groups based on their confirmatory test results (Table 2). It is also worth noting that patients who did not achieve 170 mmol/24 hours of urinary sodium excretion had similar use of angiotensin type II receptor blockers, angiotensin-converting enzyme inhibitors, and diuretics to those who did. Moreover, patients were explicitly instructed to take the sodium chloride tablets at a dose of 6 g per day. It is therefore reasonable to conclude that this does not explain the discrepancy in diagnostic performance observed in favor of OSLT compared with SSIT.

Given the considerable prevalence of PA in patients with hypertension, the widespread adoption of screening tests has led to a significant increase in the demand for confirmatory tests [39]. Consequently, there has been a corresponding increase in the expenditure associated with these diagnostic procedures. Comparing the economic aspects of both tests, in our center, the cost of SSIT is approximately 307.62 CA$ (total of 26 762.94 CA$ for our total cohort of 87 patients) and the costs associated with OSLT are 24.20 CA$ (total of 2 105.40 CA$ for our total cohort of 87 patients). Considering that if 1 confirmatory test is normal, a different test must be performed, since 46 patients in our cohort had an abnormal OSLT (and 41 patients would require confirmatory SSIT), an average cost of 134.83 CA$ would have been saved per patient (considering the need of repeated urinary collections in 14% of our cohort). From a resource allocation perspective, the use of OSLT as a first-line diagnostic test would free up nursing staff and a seat in the dynamic testing center. In our study, both SSIT and OSLT were safe and generally well tolerated. A small proportion of patients experienced a significant increase in blood pressure, although this was easily managed in all cases.

### Study Limitations and Strengths

The principal limitations of the study were the relatively small number of participants, especially considering the subgroup analysis and the retrospective nature of the study, which may be associated with selection bias, and the selection of patients only with DRC <8 ng/dL. Also, because the 24-hour urine samples were collected on an outpatient basis, their accuracy may be questioned, but this reflects a real clinical issue. Although the accuracy of urine collection relied on the individual patient, our 24-hour creatinine measurements were within the expected range to confirm adequate collection in 97% of patients. The 2 patients with small deviation in their 24-hour urinary creatinine were included in the study because they fell within the expected standard error.

Another potential limitation relates to the diagnostic standard used in our study. This explains the 100% specificity for both tests, since our study included only patients with a high pretest probability of overt PA and the fact that the diagnosis in our study depended on having 1 of the 2 confirmatory tests abnormal. However, considering all abnormal OSLT and/or SSIT results as confirming the diagnosis of overt PA is in line with the latest clinical guidelines [11]. The absence of definitive diagnostic gold standard for overt PA is a limitation of our study design but reflects a clinical reality.

The strengths of the study are the use of LC-MS/MS for the measurement of PAC and urinary aldosterone, which significantly improves the specificity of biochemical analysis, and its relevance by comparing the diagnostic performance of 2 commonly used and available methods, considering clinically relevant thresholds. To our knowledge, this is also the first study to compare the sensitivity of OSLT with SSIT in the diagnosis of overt PA.

### Conclusion

In conclusion, when performed carefully the OSLT appears to be a superior and practical alternative to the SSIT for the diagnosis of overt PA according to current guidelines using clinically relevant and previously published thresholds. This may be explained by the fact that the OSLT integrates multiple influences of postural changes and possibly other abnormal stimuli over a 24-hour period. In this context, our results suggest that a 2-step confirmatory process, starting with OSLT and followed by SSIT or another confirmatory test in the case of an initial normal result and a high clinical index of suspicion may detect more cases of overt PA at lower cost and freeing up medical resources.

## Data Availability

Some or all datasets generated during and/or analyzed during the current study are not publicly available but are available from the corresponding author on reasonable request.

## Abbreviations

ACTH: adrenocorticotropin
ARR: aldosterone–renin ratio
AVS: adrenal vein sampling
BA: both abnormal
CCT: captopril challenge test
DDD: defined daily dose
DRC: direct renin concentration
LC-MS/MS: liquid chromatography coupled to tandem mass spectrometry
NPV: negative predictive value
OSLT: oral sodium loading test
PA: primary aldosteronism
PAC: plasmatic aldosterone concentration
PPV: positive predictive value
RAS: renin–angiotensin system
SSIT: seated saline infusion test
